# Nanoelectromechanical Tuning of High-*Q* Slot Metasurfaces

**DOI:** 10.1021/acs.nanolett.3c00999

**Published:** 2023-06-12

**Authors:** Tianzhe Zheng, Hyounghan Kwon, Andrei Faraon

**Affiliations:** T. J. Watson Laboratory of Applied Physics and Kavli Nanoscience Institute, California Institute of Technology, 1200 East California Boulevard, Pasadena, California 91125, United States

**Keywords:** Light, Metasurfaces, Slot waveguide, Nanoelectromechanical systems, Resonant structure

## Abstract

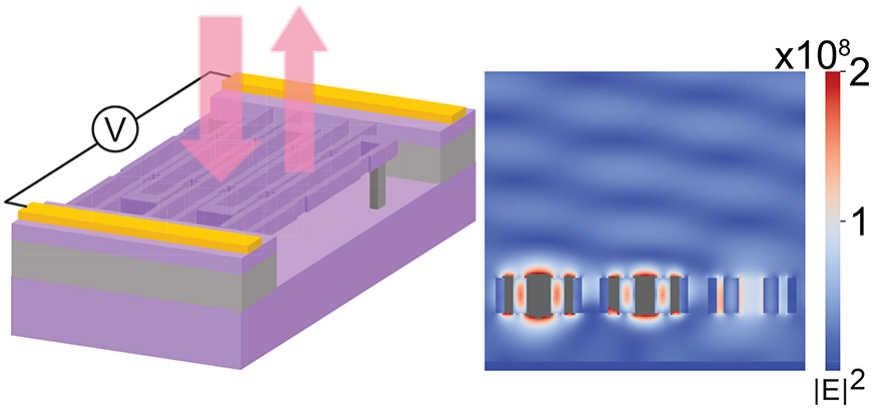

Nanoelectromechanical devices have been used widely in many applications across photonics, electronics, and acoustics. Their incorporation into metasurface systems could be beneficial in designing new types of active photonic devices. Here, we propose a design of active metasurfaces using a nanoelectromechanical system (NEMS) composed of silicon bars which operates under CMOS-level voltage and achieves phase modulation with wavelength-scale pixel pitch. By introducing a perturbation to the slot mode propagating between the silicon bars, the device operates in a high-*Q* regime, making the optical mode highly sensitive to mechanical movement. An over 12 dB reflection modulation is observed by full-wave simulation, and over 10% is achieved in the proof-of-concept experiment under CMOS-level voltage. We also simulate a device with 1.8π phase response using a bottom gold mirror. Based on this device, a 3-pixel optical beam deflector is shown to have 75% diffraction efficiency.

Metasurfaces composed of subwavelength scatterers have shown novel optical properties and scalability thanks to compatibility with mature semiconductor nanofabrication technology.^1^ Capabilities for controlling phase,^2^ polarization,^3^ and amplitude^4^ using a thin layer of artificially designed nanostructures have been demonstrated. This has led to numerous applications such as lenses,^5^ holograms,^3^ and spectrometers^6^ and unveiled the potential of compact but multifunctional optical devices.^7^

However, most metasurfaces have static optical responses defined through designs, which do not meet the need for dynamic manipulation of optical properties. Achieving spatiotemporal light control with subwavelength spatial resolution is still an outstanding goal in photonics research.^8^ Therefore, different tuning mechanisms to achieve metasurfaces with reconfigurable optical modulations in amplitude, phase, or polarization^8^ have been investigated. For example, carrier injection in transparent conducting oxides has been shown to achieve efficient phase tuning.^9,10^ However, the use of the epsilon-near-zero regime causes large absorption, and thus the overall efficiency is low. Phase-change materials^11,12^ achieve large refractive index tuning Δ*n* ≈ 1, but the intrinsic material properties limit both continuous index tuning and endurance.^13^ Recently, it was shown that organic electro-optic materials^14,15^ with record-high *r*_33_ could also achieve large refractive index tuning with GHz speed. However, the high operation voltage and stability in ambient conditions remain challenges for a wide range of applications. Nanoelectromechanical systems (NEMS) or microelectromechanical systems (MEMS)^16,17^ use electrostatic force to induce the mechanical movement of nanoscale/microscale components. They have the advantage of low power consumption, CMOS integration, and low cost.^18,19^ Over the past years, NEMS/MEMS systems combined with photonic structures^20−23^ have shown the great potential of active photonic platforms, but beam steering in wavelength-scale pixel size within CMOS-level voltage has not been realized yet.

In order to demonstrate active photonic devices operated at CMOS-level voltage, it is advantageous for the NEMS structure to operate using a high-*Q* optical resonance. Recently, high-*Q* resonant metasurfaces have been demonstrated in various systems.^24−26^ By breaking the symmetry of a bound state, the radiation channel of the mode can be controlled by the perturbation strength, and thus arbitrary *Q* could be achieved. This high-*Q* perturbation paves the way to further reduce the driving voltage of the nanoelectromechanical metasurfaces. Moreover, we findthat perturbing the slot mode leads to a larger sensitivity of the optical modes with mechanical movements. Here, we theoretically and experimentally demonstrate a nanoelectromechanical metasurface using a perturbed slot waveguide, which leads to high tunability under CMOS voltages. Over 12 dB and over 10% modulations are observed in simulation and experiment, respectively. Thanks to the locally resonant nature of the slot mode, the devices can locally control the optical response at the level of one slot. To showcase this property, we demonstrate a 3-pixel beam deflector with 75% diffraction efficiency.

The slot modes represent the eigenmodes where the electric field is confined within the low refractive index media between the high refractive index media.^27^ When the dimension of low refractive index media is small, the electric field will be greatly enhanced due to the discontinuity of the high-refractive-index-contrast interfaces. Over the last two decades, the slot modes have been widely explored,^28,29^ which enabled applications in sensors^30^ and modulators.^31^ However, the slot modes have been rarely used in free-space optics since they cannot couple to the free-space wave due to momentum unmatching. When periodic perturbations are applied to the waveguide, the guided mode starts to couple with the free-space light.^24,25^ To illustrate the proposed concept, we first consider an infinite number of silicon bars in air, periodically placed in the *x*-direction and infinitely long in the *y*-direction. The two bars within a period are close to each other to form a slot. A period of the structure is drawn in Figure 1a, including the perspective view and relevant cross sections. Notches are introduced in the design in order to create a periodicity in the *y* direction and wrap the dispersion relation of the slot modes. A typical dispersion relation is shown in Figure 1b, where the periodicity is introduced fictitiously (i.e., there are no notches) in the periodic boundary conditions of the simulation. Two degenerate slot modes emerge at *k*_∥_ = *k*_*y*_ = 0. The corresponding mode profile is plotted within Figure 1b, indicating that two guided waves form a standing wave mode under this fictitious periodic condition, matching the *k*_∥_ = *k*_*y*_ = 0 momentum of the incident plane wave propagating along −*z*. Thus, when we create notches at this fictitious period *p*, the normally incident light will start to couple into the slot mode. As a result, the high-*Q* resonance will appear near the frequency of the degenerated slot mode. It is worth noting that similar designs were proposed before,^32^ but our main purpose here is to utilize these defect modes to achieve high-*Q* resonance in the NEMS system. When the notch depth increases, the perturbation strength increases, thus decreasing the quality factor. Figure 1c shows the reflection spectra of the slot resonances for several different notch sizes, and Figure 1d summarizes their *Q*-factors.

**Figure 1:**
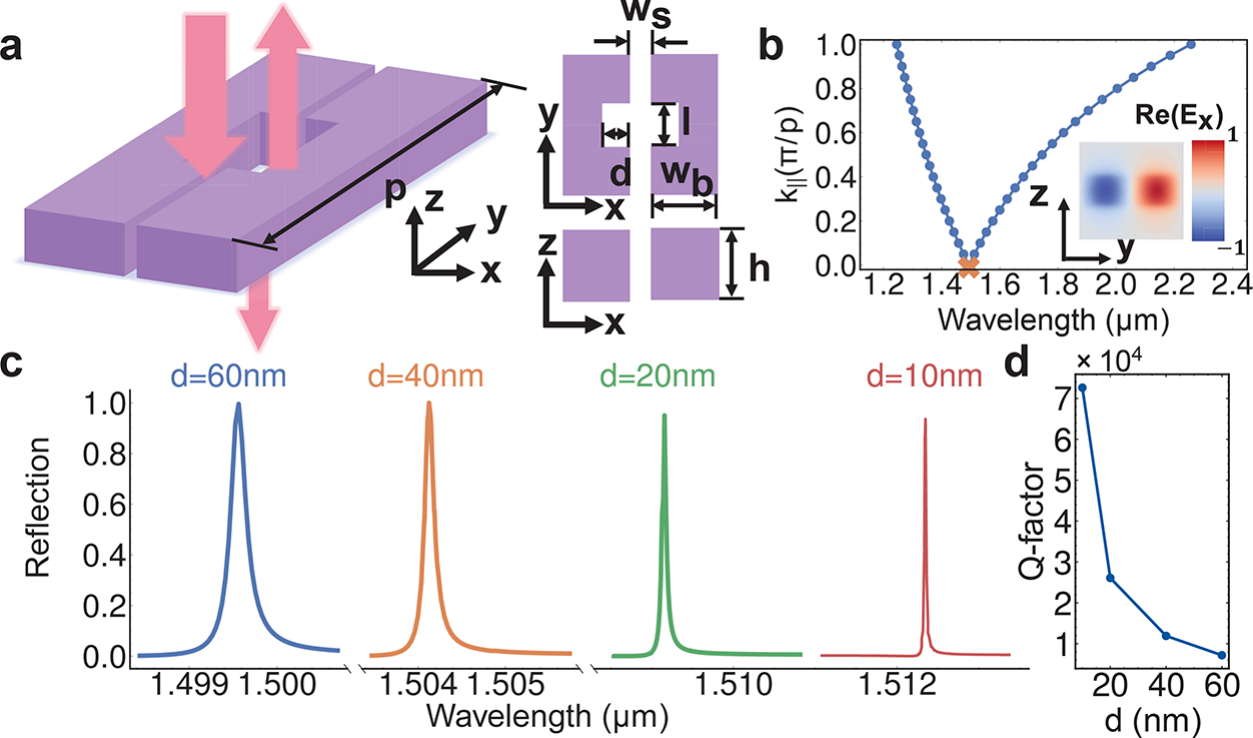
Slot mode characteristics. (a) Geometric diagram of a pair of infinite-long slots with periodic notches. Left: perspective view. Top right: top view. Top bottom: front view. The definitions of design parameters are noted: *p* is the period of the notches; *d* and *l* are the width and length of the notches; and *h* is the height of the silicon bar. *w*_s_ is the slot width between the bars with different voltages, and *w*_g_ is the gap between the bars with the same voltages. (b) The dispersion relationship of slot modes when *d* = *l* = 0. The electrical field profile represents the mode marked with the orange cross. In this figure *w*_b_ = 220 nm, *w*_s_ = 90 nm, and *h* = 500 nm. (c) Numerically calculated reflection spectra of the slot resonances. The silicon is suspended in the air without any substrate. Four different spectra are plotted by changing *d* from 10 to 60 nm. (d) Dependence of the *Q* factor on the perturbation parameter *d* for the spectra shown in (c).

Since the electrical field of the slot mode is mostly confined in air, the mode is highly sensitive to the air gap width. As a result, the horizontal movements between nanobars induced by electrostatic force will lead to significant modulation of optical signals. Figure 2a shows the conceptual schematics of the proposed NEMS-tunable devices. The system consists of two groups of suspended silicon bars in a comb-shaped arrangement. Every bar has periodical notches to create high-*Q* resonances, and every pair of nanobars is interdigitally connected to different islands for electrical biasing. To enhance the robustness, the two bars with the same voltages are linked together at the anchors.^21^ The device could be directly fabricated using a commercial silicon-on-insulator (SOI) wafer. The voltage setting and the cross-section of the system are shown in Figure 2b. One period unit with width *w*_p_ includes one pair of nanobars grounded and at a voltage separately. The voltages on the nanobars are doubly interdigitated such that when there is a voltage difference between the neighboring nanobars the electrostatic force will cause smaller *w*_s_. In the experiment, the length of the nanobars could be tens of μm and is much longer than the wavelength. Therefore, we assume that the nanobar is infinite in the *y*-direction in numerical simulations.^21,33^ The reflection spectra for different slot widths are plotted in Figure 2c. As *w*_s_ gets smaller, the resonance shows a larger amount of the redshift. To illuminate the relationship between the bias voltage and the maximum displacement, we simulate the mechanical displacement under the different voltages, assuming that the length of the nanobars is 33 μm (using Comsol Multiphysics, see Supporting Information section 1). In Figure 2d, we plot simulated results of the mechanical movement at the center of the nanobar and the absolute reflection at input wavelength λ = 1.4573 μm (where *R* = 0.05 when *w*_s_ = 80 nm) and λ = 1.4544 μm (where *R* = 0.05 when *w*_s_ = 90 nm) as a function of the applied voltage. It is worth noting that we assume the slot gaps shrink uniformly along the *y* direction in the reflection simulation, and the displacement is the same as the center of the nanobar (see Supporting Information section 1). Under the conditions above, the numerical pull-in voltage is only 1.65 V due to the small *w*_s_ of 90 nm. The maximum slot width change Δ*w*_s_ of each nanobar has an approximately quadratic relationship with the bias voltage, and within 1.5 V, the maximum displacement for each bar is over 10 nm. Thanks to the high sensitivity of the slot resonance, the reflection amplitude will have 11.9 dB modulation from 0 V to 0.926 V and 12.3 dB modulation from 1.117 V to 1.296 V. The results indicate that a high extinction ratio could be accomplished within the CMOS-level voltage with the proposed device.

**Figure 2:**
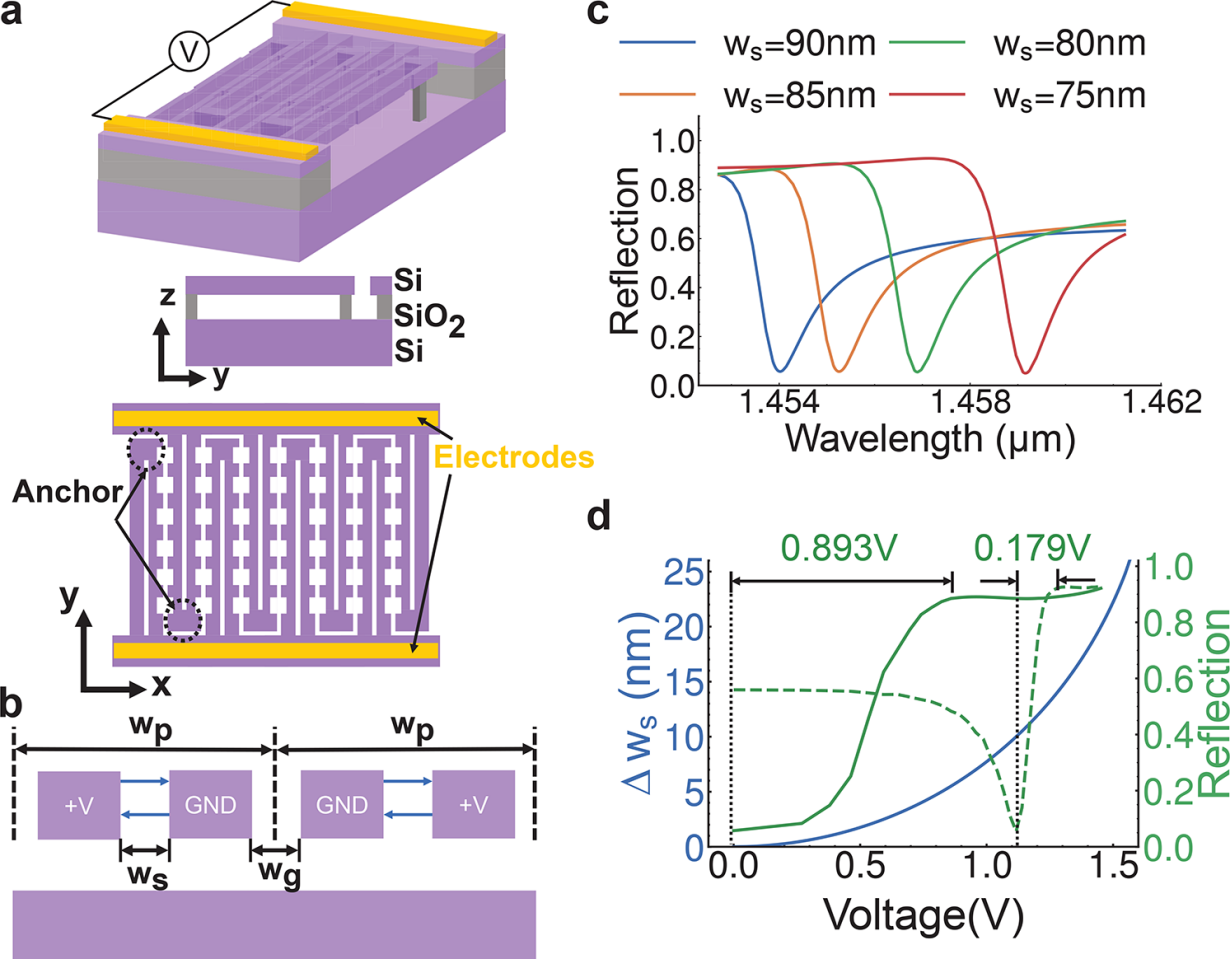
Slot mode tuning properties. (a) Device design. From top to bottom: perspective view, a cross-section along the bar, and top view. (b) The voltage setting of the device. Grounded bars (GND) and bars at a voltage (V) are interdigitally connected in groups of two. *w*_p_ is the smallest period of the device. (c) Calculated reflection spectra of the device in (a) for different spacings between nanobeams (*w*_s_). Light is incident from +*z* to −*z* direction. (d) Blue: Simulated slot gap width change Δ*w*_s_ at the center of the nanobar for different bias voltages. Solid green: Modulation of the reflection amplitude at a wavelength of 1.4544 μm. Dashed green: Modulation of the reflection amplitude at a wavelength of 1.4573 μm.

To experimentally demonstrate the proposed concept, the devices described in Figure 2a are fabricated and characterized. The fabrication methods are similar to what we previously reported.^21^Figures 3a–d show the step-by-step zoomed-in image from an optical camera image to the scanning electron microscope (SEM) image. In order to realize a suspended structure in the experiment, we implement two modifications to the theoretical design (see Supporting Information note 1). First, to make sure the anchor is fixed, we carefully control the hydrofluoric acid etching time when releasing the silicon nanobars. As a result, the BOX layer is not fully etched. Second, to improve the robustness of the device, we employ a series of anchors to extend the overall length of the slot. In Figure 3c, the device length is 150 μm, and the distance between the anchors is 25 μm. The devices are connected to two electrode pads in parallel for multidevice fabrication and testing. All electrodes are wire-bonded to a customized PCB board connected to the voltage source. We find that the experimental reflection response (Figure 3f) reproduces the simulation results shown in Figure 3e. The varied background reflection indicates a low-*Q* guided mode, and different Fano resonance shapes could be attributed to the different coupling strength between the slot mode and guided mode.^34^ Since the reflection spectra are normalized using the reflection from the gold electrode, the actual reflection should be a few percentages higher. When voltage is applied, the resonance exhibits a redshift as shown in Figure 3g. A 1.2 V bias voltage induces a 0.9 nm redshift of the slot mode, confirming that the resonance is highly sensitive to the slot gap change. Besides the redshift, the modulation of the reflection is 10% at λ = 1.569 μm. In addition to the redshift, there is also an amplitude decrease, which we attribute to diminished coupling between the slot modes and the incident beam.^35,36^ When the electrical bias is applied, the bending of the two-sided fixed suspended bars occurs, causing local slot width variation along the *y* direction. The local variation in slot widths will decrease the local coupling rate between the incident light and the slot mode.

**Figure 3:**
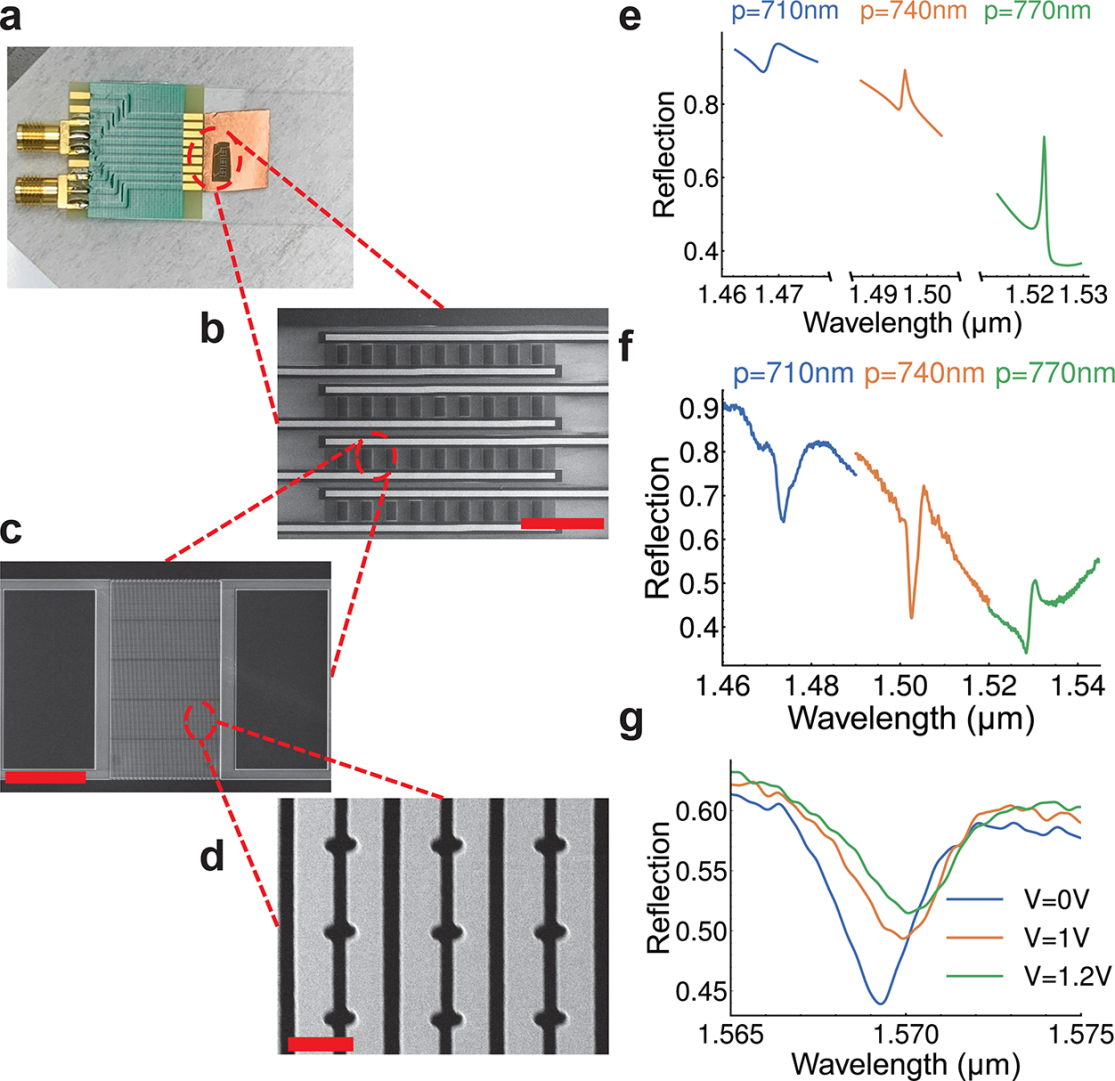
Experimental NEMS tuning. (a) An optical image of the chip that is wire bonded to a customized print circuit board. (b)–(d) Scanning electron microscopy images of the device. The scale bars are 500 μm, 50 μm, and 500 nm, respectively. (e)–(f) The simulated (top) and experimental (bottom) reflection spectra of the proposed device for different notch periods *p*. (g) The reflection spectra of the slot metasurface devices under different electrical bias voltages.

Although large amplitude modulation could be achieved through the aforementioned designs, the phase response is still limited due to the limited coupling coefficient from the input plane wave to the resonant mode.^9,37^ To enhance the phase response, one of the potential solutions is to add a mirror at the bottom of the nanobars.^9,10^ The mirror reflects the light and enhances the coupling between the resonant slot mode and the illuminated light from the top. Here, we propose a NEMS design with a metal mirror, where the phase response could be close to 2π.

The cross-section of the design is presented in Figure 4a. The gold mirror is added between the oxide layer and the silicon substrate. To introduce a fabrication-compatible structure and simplify the discussion, we assume that the silicon oxide layer is fully etched when releasing the silicon bars. Except for the anchor regions on both sides of the bars, there are only air gaps between the silicon nanobars and the gold mirror. This structure could be fabricated by wafer bonding and thinning,^38,39^ and the gold mirror could also be replaced by a Bragg mirror.^37^ The existence of the gold mirror will enhance the mode coupling with the upper port, as the mirror reflects the light upward. Thus, the reflected phase response at the resonance will get enhanced to nearly 2π. Figure 4b shows the reflection amplitude and phase around the slot resonance for different slot gaps *w*_s_. Due to the high reflectivity of the gold mirror, when the device is off-resonant the overall reflectivity is close to 1. The coupling between the incident wave and slot mode resonance will induce a Fano reflectivity line shape.^40^ The phase response covers 1.8π from *w*_s_ = 70 nm to *w*_s_ = 90 nm. The high reflectivity and large phase coverage indicate that the resonance is highly overcoupled. To illustrate the effect of an air gap over the coupling strength, the complex reflection coefficients for slot resonances at different air gaps *h*_a_ are plotted in Figure 4c. Each resonance generates a circle in the complex plane. As *h*_a_ increases, the area of the circle also increases, indicating the increase of the coupling strength.^37^ At *h*_a_ = 275 nm, the zero reflectivity shows that the critical coupling regime is achieved. Further increase of *h*_a_ will help the mode resonance enter the overcoupling regime, where the circle includes the origin point. At *h*_a_ = 350 nm the phase coverage is close to 2π, and the overall reflection is over 80%. This indicates that it is possible to achieve phase-only tuning from slot mode resonance. As an example, Figure 4b shows that when we change the slot width we could achieve 2π phase-only modulation with nearly unity reflection.

**Figure 4:**
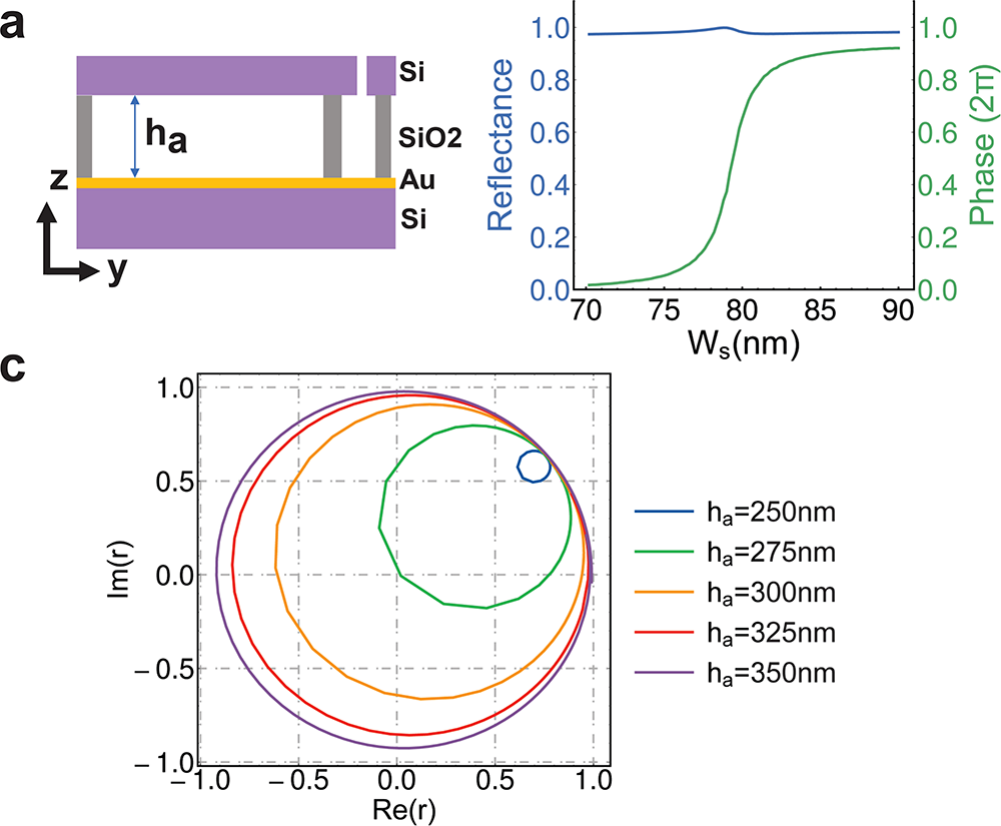
Design of phase modulator. (a) The cross-section of the proposed structure that incorporates nearly 2π phase modulation. (b) The reflection amplitude and phase around the slot resonance as a function of different slot sizes. By reducing the slot gap size from 90 nm to 70 nm, the reflectance remains over 97%, while the phase coverage is over 1.8π. (c) The reflection coefficient in the complex plane around the slot resonance in air gap thicknesses *h*_a_ from 250 nm to 350 nm. When *h*_a_ > 275 nm, a phase coverage close to 2π is achieved.

Another property of the slot mode resonance is that the mode remains locally resonant within the slot. The combination of the locally resonant slot mode and enhanced 2π phase modulation makes it possible to achieve one-dimensional spatial phase modulation at the wavelength-scale pixel level. To unveil the potential of wavefront engineering on this platform, we design an active beam deflector which can attain diffraction efficiency of ∼75% in the first order. The design for one period is illustrated in Figure 5a. It should be noted that although the slot resonance is mostly confined within the slot, the crosstalk between the adjacent slots can only be ignored in the amplitude-related design but remains crucial in phase-related design (see Supporting Information sections 3 and 4). To enable the spatial tuning of the slot metasurfaces, the cross-coupling between the adjacent slots should be blocked. We use a small silicon nanobar to block the cross-coupling,^41,42^ and the gaps between the nanofins *w*_aw_ and the nanofin width *w*_bw_ are chosen to be only 100 nm to reduce the pixel size but keep the fabrication compatibility. The existence of the additional adjacent nanofins will extend the period length to 1200 nm, but the size of the pixel is still in the subwavelength regime in the air. These additional nanofins will cause a small redshift of the resonance frequency, but the phase coverage is preserved, as shown in Figure 5b. From this one-to-one mapping relationship between slot size *w*_s_ and phase response, we pick 3 points (0.55π, 1.27π, 1.97π) that are roughly spaced by  to form a 3-pixel supercell structure, labeled as blue stars. The 3 different points are intentionally chosen to be larger to avoid the pull-in effect, and they are optimized around the exact  spacing points due to the residual crosstalk between different slots. As shown in Figure 5c, the diffraction efficiency achieves a maximum of ∼75% in 1.504 μm for the first order. Figure 5d illustrates the electrical field profile of the slot structure at 1.504 μm, where the first-order steering angle is 24.7°. Since the slot gap size change is only ∼30 nm, the required voltage to drive the beam steering should be at the CMOS level.

**Figure 5:**
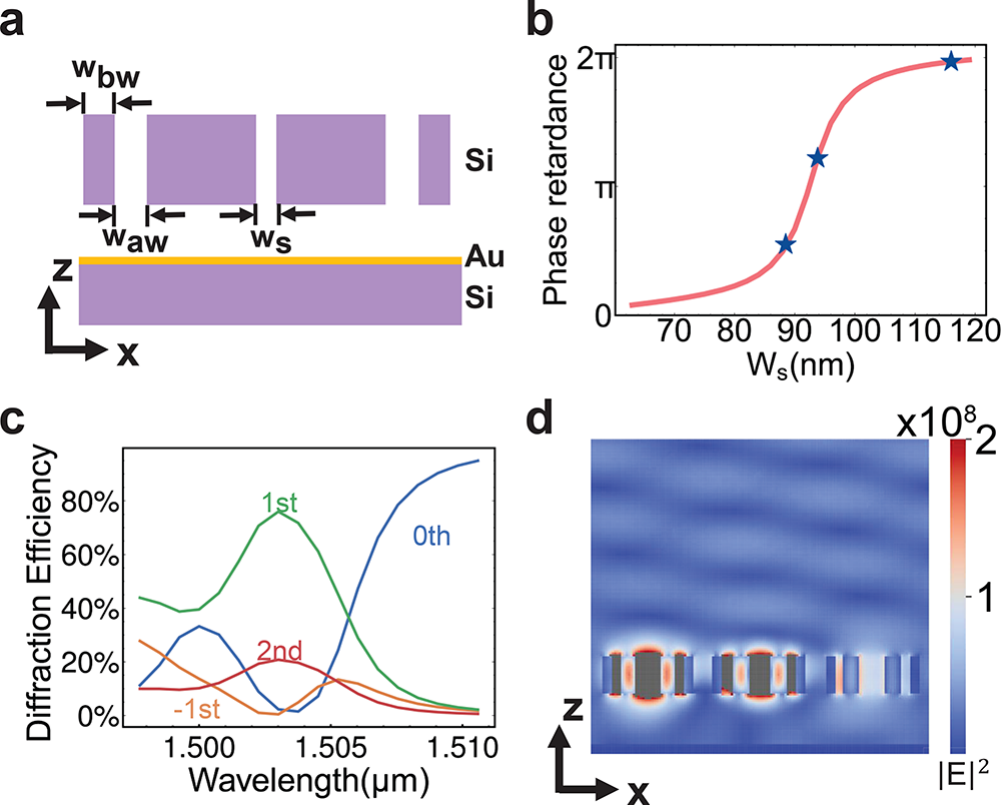
Demonstration of a beam deflector. (a) The geometry of one periodic unit of the beam deflector. The nanobar blockers with width *w*_bw_ are added to prevent the cross-coupling between the pixels. The nanobar blockers do not have notch perturbation. (b) The phase response of the slot mode with respect to the slot size *w*_s_. The blue stars represent points where *w*_s_ = 88.5 nm, 93.8 nm, and 116 nm, respectively. We choose these 3 slot gaps to form a 3-period supercell beam deflector. (c) The diffraction efficiency of the device designed according to (b). The major diffraction orders (−1^st^, 0^th^, 1^st^, 2^nd^) are shown. (d) The electric field profile of the device described in (b) at 1.503 μm. The gray pixels indicate that the field is saturated.

We note that the beam deflector design shown in Figure 5 could be easily extended to supercells including more slots for the purpose of smaller deflection angle and ultimately quasicontinuous beam steering.^9,10^ However, our computing tools do not allow for simulating these larger structures because of lack of memory. Although using the phase gradient is the easiest way to generate a steered beam, advanced optimization techniques are expected to result in suppressed side lobes and enhanced directivity.^43^

In conclusion, in this letter we propose a platform that could achieve efficient amplitude and phase tuning under the CMOS-level modulation voltage and have a wavelength-scale pixel level. It could achieve 16.9 dB modulation within 0.752 V in numerical simulation. We also observe 10% reflection modulation under 1.2 V bias voltage in the experiment. The locally resonant property enables individual reflective phase tuning in individual slots. With a more judicious design, it achieves 75% diffraction efficiency. The key component of the platform is that the slot mode resonance is highly sensitive to electrostatic perturbation thanks to the confined electric fields in the slots.

For the future directions, the required driving voltage may be further reduced by inverse-designed gratings, as we believe more sophisticated nanostructures could lead to a stronger optical response to mechanical displacement. By utilizing the avoided crossing of resonances, the phase modulation capacity could be potentially improved up to 4π.^44^ Furthermore, a more complicated phase profile could be generated if the number of pixels is extended. We expect that the combination of the slot modes and the NEMS platform will enable 1D high-resolution phase-only spatial light modulators with CMOS-level operations, lower power consumption, and scalable manufacturing.

## Supporting Information Available

Additional information about the designs and experiments, including simulation and experiment details, the dispersion of slot modes, multiwavelength resonant device design, the crosstalk between the slots, and the AC analysis (PDF)
