# Supplementary material for: Nanoelectromechanical Tuning of High-Q Slot Metasurfaces
Source: Nano Lett. 2023 Jun 12;23(12):5588–94. doi: 10.1021/acs.nanolett.3c00999 (PMC10311603; doi:10.1021/acs.nanolett.3c00999)
Supplement: Supplementary file 1 — nl3c00999_si_001.pdf [file nl3c00999_si_001.pdf]

Supplementary information: Nanoelectromechanical tuning of high-Q slot Metasurface

Tianzhe Zheng,[1] Hyounghan Kwon,[1,2,*] and Andrei Faraon[1,2,†]

[1] *T. J. Watson Laboratory of Applied Physics and Kavli Nanoscience Institute, California Institute of Technology, 1200 E. California Blvd., Pasadena, CA 91125, USA*

[2] *Department of Electrical Engineering, California Institute of Technology, 1200 E. California Blvd., Pasadena, CA 91125, USA*

* Current address: Center for Quantum Information at Korea Institute of Science and Technology, Address. 5, Hwarang-ro 14-gil, Seongbuk-gu, Seoul, South Korea

† Corresponding author: A.F.: faraon@caltech.edu

SUPPLEMENTARY NOTE 1: SIMULATION AND EXPERIMENT DETAILS AND DETAILED DEVICE PARAMETERS

The optical simulation is performed using COMSOL Multiphysics™with periodic conditions applied along the x and y directions (see Fig. 1 in the main text). Detailed simulation device parameters are provided in Table S1.

For the mechanical simulation shown in Fig. 2d, we also employ COMSOL Multiphysics™. The nanobar length is set to $33\mu m$, with both ends fixed. The nanobars serve as terminals with a fixed voltage V or ground. We calculate the displacement as the maximum displacement along the x-direction at the center of the nanobar. The displacement profile, shown in Fig. S1, exhibits a bell curve with maximum displacement at the center. Specifically, for a nanobar length of $33\mu m$, the regions where displacements exceed 80% and 50% of the maximum displacement are $11\mu m$ and $18\mu m$, respectively. In Figures 3c-d, we assume a uniform shrinkage of w_s for simplicity. Considering the relatively flat displacement curve at the center of the nanobar ($y = 0$), we also assume that the displacement is the same at the center along the y-direction. Furthermore, the decrease in resonance amplitude observed in Figure 3g can be explained by the non-uniform property of the mechanical displacement.

In Fig. 2a, the silicon oxide layer beneath the suspended nanobars is fully etched. In the experiment, we create an air gap and release the nanobars using hydrofluoric (HF) acid etching. To fix the anchor while releasing the nanobar, we carefully control the etching time. In our experiment, the silicon-on-insulator (SOI) wafer has a $3\mu m$ buried oxide (BOX) layer thickness, resulting in a remaining oxide layer after nanobar release. The thickness of the remaining oxide layer, h_o, is indicated in Table S1. We note that the surface of the remaining BOX layer is non-uniform due to the isotropic nature of HF etching. The estimated remaining layer thickness is based on the estimated rate of HF acid etching. For the designs in Fig. 2, Fig. 4, and Fig. 5, we do not consider these experimental details and assume $h_o = 0$.

In the simulation, we assume an infinitely long slot length and apply periodic boundary conditions along the y-axis. However, in the experiment, the length of the nanobars plays a crucial role in validating this approximation and determining the speed (see supplementary note 5). Since the distance between the nanobars is only approximately 90 nm, increasing the length of the slot makes it susceptible to perturbations during fabrication. To further extend the slot length, we incorporate additional anchors between the air gaps adjacent to each slot. These anchors connect

the adjacent nanobars, and their length is chosen to be sufficient for connecting the anchor to the unetched silicon oxide layer. To strike a balance between the minimum bias voltage required for tuning and the fabrication yield rate, we choose an anchor distance of $w_y = 25\mu m$. The top view of the experimental fabricated device is illustrated in Figure S2, showcasing the extended slot length achieved through this approach.

The measurement setup is depicted in Figure S3. Detailed information about the setup and experimental procedures can be found in our previous report [1]. To obtain the reflection spectra of the x-polarized input light, the grating is aligned at 90 degrees with respect to the beam splitter axis. The polarizer (Pol.) and the half wave plate (HWP) in front of the objective lens are aligned at 45 and 22.5 degrees, respectively.

Device	p	h	w_p	w_s	w_{b1}	w_{b2}	d	l	h_a	h_o	w_{aw}	w_{bw}
Figure 1b-d	varied	500	700	90	220	220	varied	varied	N/A	N/A	N/A	N/A
Figure 2c	740	490	700	varied	240	240	60	110	150	0	N/A	N/A
Figure 3e-f	varied	490	1400	90	555	555	90	120	250	2700	N/A	N/A
Figure 3g	870	490	740	90	245	245	70	120	300	2700	N/A	N/A
Figure 4b	720	500	800	90	250	250	60	90	500	0	N/A	N/A
Figure 4c	720	500	800	90	250	250	60	90	varied	0	N/A	N/A
Figure 5b	720	500	1200	90	250	250	60	90	650	0	100	100
Figure S5	590	570	1000	90	345	335	60	80	150	0	N/A	N/A

Table S1 Device parameters used for the plot in main figures (unit:nm) N/A: not applicable. Varied: the value of the parameter is a variable in main figure.

SUPPLEMENTARY NOTE 2: FURTHER DISCUSSION ON THE SLOT MODE DISPERSION

Figure S4 presents the electrical field profiles of additional points in Figure 1b from the main text. At $k_{||} = k_y = 0$, two degenerate slot eigenmodes are observed, as shown in Figure S4(b) and (c), respectively. These profiles exhibit a $\pi/2$ phase offset along the y-axis, indicating standing wave modes. The formation of these standing waves arises from the degeneracy of the slot modes at $k_{||} = 0$. Under this fictitious periodic condition, two bands converge at the same wavelength, $\lambda = 1.493\mu m$, and the structure supports two orthogonal slot eigenmodes represented by orthogonal standing wave modes.

When $0 < k_{||} < 1$, the eigenmodes split into two branches at longer and shorter wavelengths, respectively. Figure S4(d) and (e) illustrate the mode profiles for longer and shorter wavelengths, specifically when $k_{||} = \pi/(2p)$ (where p denotes the period of the fictitious perturbation, as shown in Figure 1b). The constant amplitude of E_x and the periodic variation of $Re(E_x)$ in both modes confirm their nature as propagating modes along the slot. Within the simulation region ($-500nm < y < 500nm$), the mode at the longer wavelength (e) exhibits more periods compared to the mode at the shorter wavelength (d), while both modes satisfy the boundary Floquet condition along the y-axis.

Finally, when $k_{||} = \pi/p$, the boundary condition is the same as $k_{||} = 0$, except for a phase shift of π. In this case, two degenerate eigenmodes can be identified at different wavelengths. Figure S4(f)-(g) and (h)-(i) display the two sets of degenerate modes, respectively. Similar to the $k_{||} = 0$ case, these modes are standing waves and are formed by two propagating modes. Overall, this fictitious periodic perturbation wraps the band and facilitates the matching of momentum between the incident light and the propagating slot mode. By introducing this periodic perturbation, we can open up the slot mode radiation channel to free-space light.

In Figure S5, we examine the field profiles of eigenmodes for different notch sizes. As the notch size increases, the field enhancement within the slot (at z = 0) decreases, explaining the observed decrease in the quality factor.

SUPPLEMENTARY NOTE 3: MULTIWAVELENGTH RESONANT DEVICE WITH INDIVIDUAL SLOT TUNING IN AMPLITUDE

In this section, we present a demonstration of individual optical tuning at two distinct wavelengths, accomplished through the utilization of a multiwavelength resonant device featuring two slot resonances. The novel design approach adopted in this study permits independent amplitude tuning of the slot mode, obviating the need for the block design illustrated in Figure 6. Figure S6a shows the proposed device designed for operations at two different wavelengths. The x-direction period includes 8 bars in total: 4 pairs of V and GND nanobars with total width w_p. Each pair hosts a slot resonance, with the slot gaps w_{s1} or w_{s2}. When $V_1 = V_2 = 0$, $w_{s1} = w_{s2}$. To create slot resonances at two different wavelengths, every two pairs have different slab widths w_{b1} or w_{b2}. For this example, $w_{b2} - w_{b1} = 10\ nm$. This device is out of the subwavelength regime since $w_{b1} \neq w_{b2}$. However, since the difference between w_{b1} and w_{b2} is only $10nm$ the structure is only weakly diffracting (See supplementary material section 3). In this voltage setting, the slot widths w_{s1} and w_{s2} could be dominantly controlled by V_1 and V_2, respectively. In other words, the control voltage V_1 (V_2) mainly shrinks the gap w_{s1} (w_{s2}) but does not affect the gap w_{s2} (w_{s1}) since the electrostatic force will mostly affect the adjacent nanobar at a different voltage. Therefore, the tuning voltage $V_1(V_2)$ can control the corresponding slot gap $w_{s1}(w_{s2})$ while keeping the other slot gap w_{s2} (w_{s1}) unchanged, and the period width remains constant. Figure S6b shows the reflection spectra when we change the w_{s1} and w_{s2} separately while keeping the other constant. We also plot the electric field profile in the corresponding resonance. The electric field profile only includes half of the period because of symmetry. In Figure S6b, the resonances in shorter (longer) wavelengths are related to the slot on the left (right), and the other slot remains non-resonant because of the frequency detuning. Since only half of the slots are excited in the resonance, the reflection modulation amplitude is limited to 50 %. Thus, the maximum amplitude modulation is 13.3 dB in 1.491 μm and 20.5 dB in 1.509 μm. The green(orange) curve indicates that when we only move $w_{s1}(w_{s2})$, the resonance in low(high) wavelength will show the redshift, but the other resonance remains almost undisturbed. As a result, this design strategy will allow us to modulate the reflectivity in multiple wavelengths independently.

The device for multiwavelength operation will have first-order diffraction. The 0th order and the 1st order spectra are shown in Figure S6 (The -1st order has the same power as the 1st order due to symmetry). When the input wave is not on resonance, the direct reflection is slightly over

10% and the first-order reflection is nearly 0, as the geometry difference between the two slots is small. On resonance, the 0th and 1st order reflective power increases to 40% and 10% respectively. The total reflective power has increased to 60% due to the increased reflection from the 0th order and 1st (-1st) diffraction channel. This confirms that when the input wave is on resonance, the reflective power in the 0th order is not coming from other reflective diffraction channels, but from the transmission channels.

SUPPLEMENTARY NOTE 4: THE CROSSTALK BETWEEN ADJACENT BARS

The added block in Figure 5a has the scope to prevent the crosstalk between slots. To prove that it is necessary to add the blocks, we also perform full-wave simulations on the slot metasurfaces without any blocks. Figure **S7**a shows the dictionary of the phase response for the periodic slot structure. We choose (0.494π,1.200π,1.876π) to form a beam steering supercell. The resulting electric field profile under the normal incident light is in Figure **S7**b. Instead of showing clear beam deflection, the profile incorporates different diffraction orders. Thus, the directivity of the beam deflection is lower, and there isn't a clear beam steering direction in the electrical field profile.

SUPPLEMENTARY NOTE 5: NOTE ON AC ANALYSIS AND MECHANICAL EIGENFREQUENCY

The mechanical eigenfrequency of the system is numerically investigated using COMSOL Multiphysics™. The simulation setting is the same as the one in supplementary note 1. By varying the distance between the anchors (fixed boundary), the eigenfrequency of the device Fig. 3g is shown in the table:

W_y	Eigenfrequency (MHz)
$25\mu m$	3.5
$50\mu m$	0.89
$75\mu m$	0.40
$100\mu m$	0.22
$125\mu m$	0.14

Table S2 Mechanical eigenfrequency of the nanobars shown in Fig. 3g assuming different distances of anchors W_y.

The AC characteristic of this NEMS platform was previously investigated in ref[1]. Since this device has a longer device length (150 μm), it is estimated that our experiment device has lower bandwidth (< 25kHz). This frequency is much lower than the mechanical resonance frequency of the system, so we will only consider the AC response without any resonant effect. We provide a simplified circuit model to explore the AC performance tradeoff of the system, shown in Fig. We assume that the circuit includes a source resistance $R = 50\Omega$, and we model the parallel nanobars as parallel resistances $Rs = \rho\frac{l_{si}}{hw_b}/N = 0.13G\Omega$, where silicon resistivity $\rho = 10\Omega \cdot m$, nanobar height $h = 500nm$, nanobar width $w_b = 220nm$,slot gap width $w_g = 90nm$, number of nanobars $N = 100$. The slot gap capacitance $Cs = C_{per}l_{si}(N-1) = \epsilon_0\frac{K(\sqrt{1-k^2})}{K(k)}l_{si}(N-1) = 0.19pF$ is modeled using the calculation from [2], where $K(k)$ is the elliptical integrals of the first kind, and $k = \cos(\frac{\pi w_b}{2w_g})$. The response of the voltage on the capacitor could be expressed as

$$V_{Cs} = V_0\left|\frac{1/(j\omega C_s)}{R_s + 1/(j\omega Cs) + R}\right| \quad \textbf{(S1)}$$

We can calculate the 3dB attenuation frequency for voltage is $f_{3dB} = 10.4kHz$.

I. REFERENCES

[1] Kwon, H., Zheng, T. & Faraon, A. Nano-electromechanical tuning of dual-mode resonant dielectric metasurfaces for dynamic amplitude and phase modulation. *Nano Letters* **21**, 2817–2823 (2021).

[2] Benea-Chelmus, I.-C. *et al.* Gigahertz free-space electro-optic modulators based on mie resonances. *Nature Communications* **13**, 1–9 (2022).

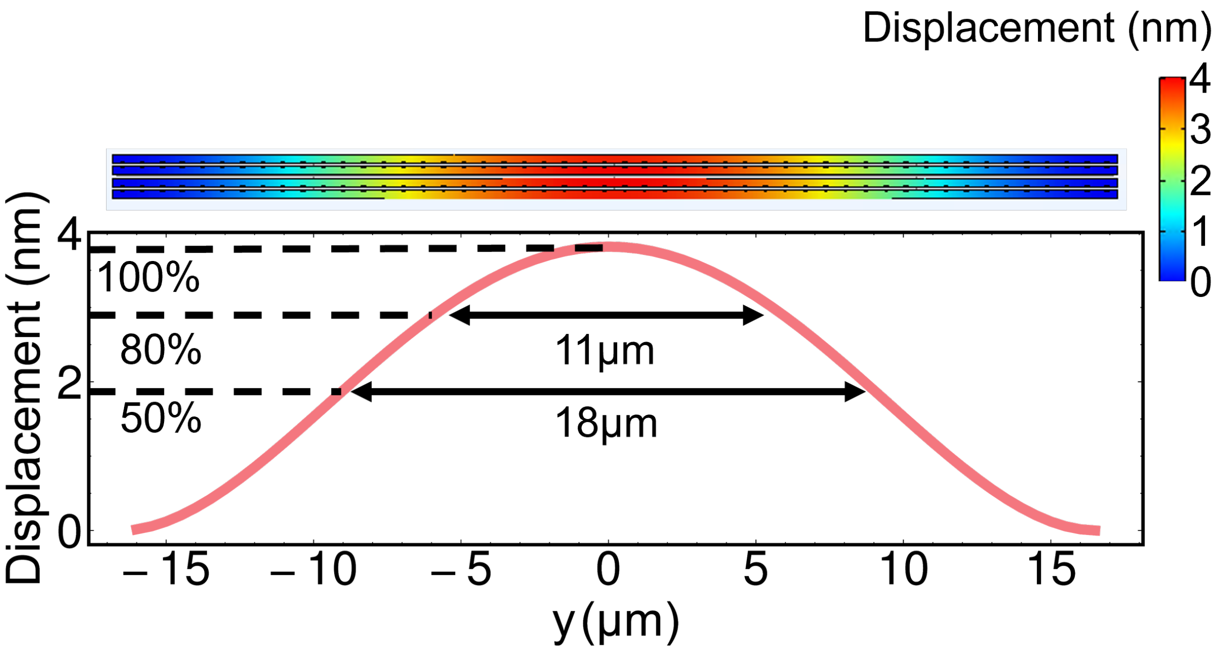

Figure S1 Mechanical displacement profile of the nanobars when $V = 1V$**.** The displacement is the absolute displacement for one nanobar. Top: the colormap of the mechanical displacement in the top view. Bottom: the 1D displacement profile along the nanobar.

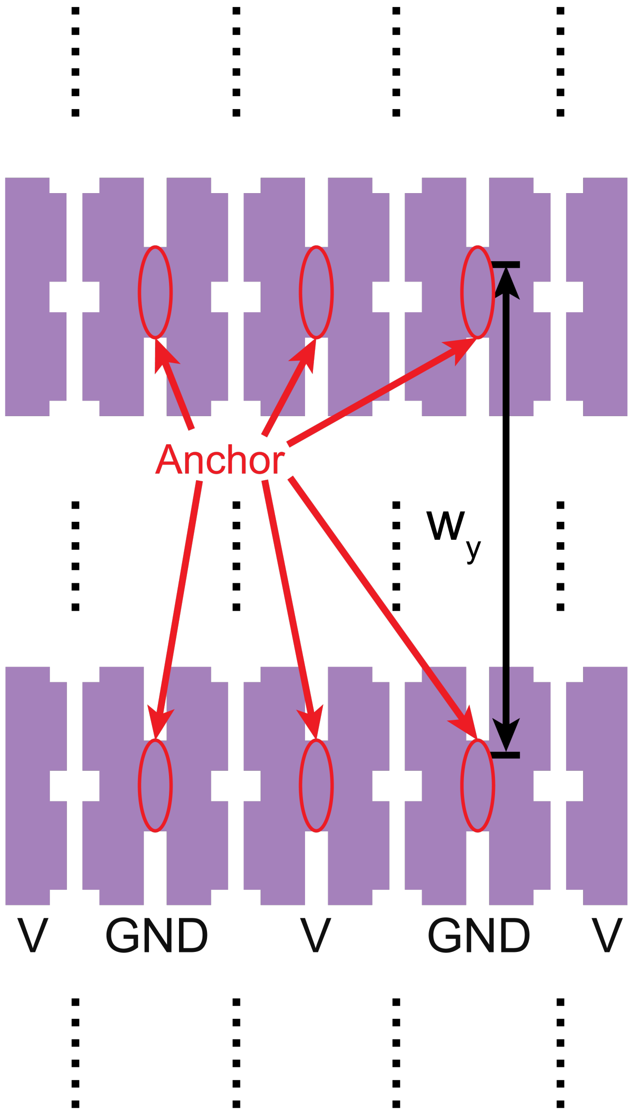

Figure S2 Top view of Experimental device with a series of anchors. The newly created anchor connects the adjacent bar with the same bias voltage. w_y: the y-axis distance between the anchors.

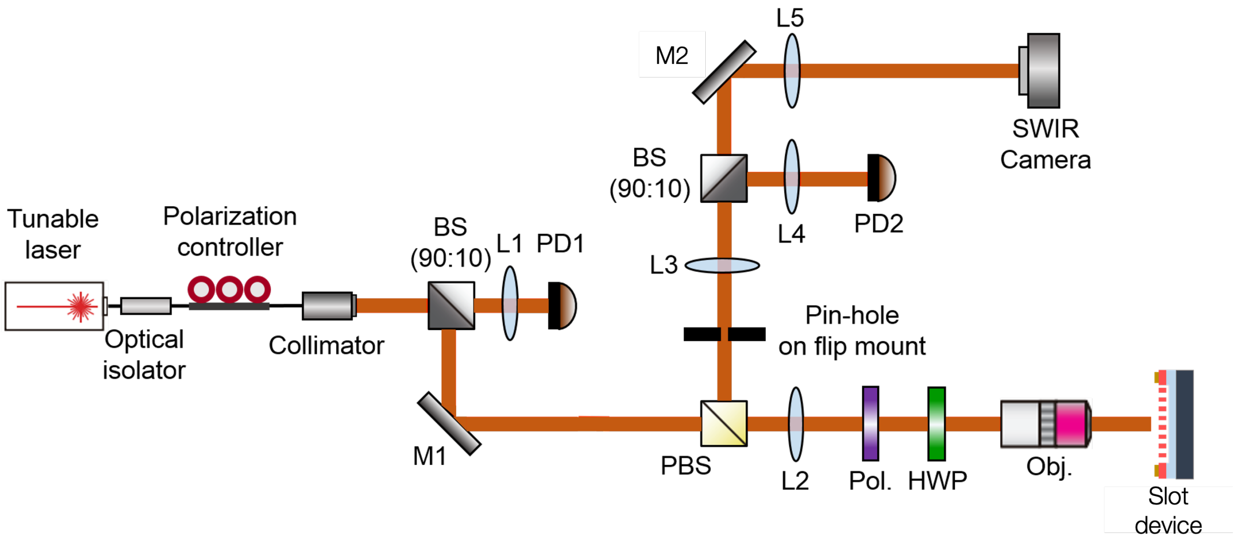

Figure S3 Schematic illustration of the experimental setup. The brown line indicates the paths of the light propagation. Pol.: linear polarizer. BS: beamsplitter. PBS: polarizing beamsplitter. L: lens. PD: photodetector. M: mirror. QWP: quarter waveplate. HWP: half waveplate. Obj.: microscope objective lens. SWIR camera: short-wave infrared camera.

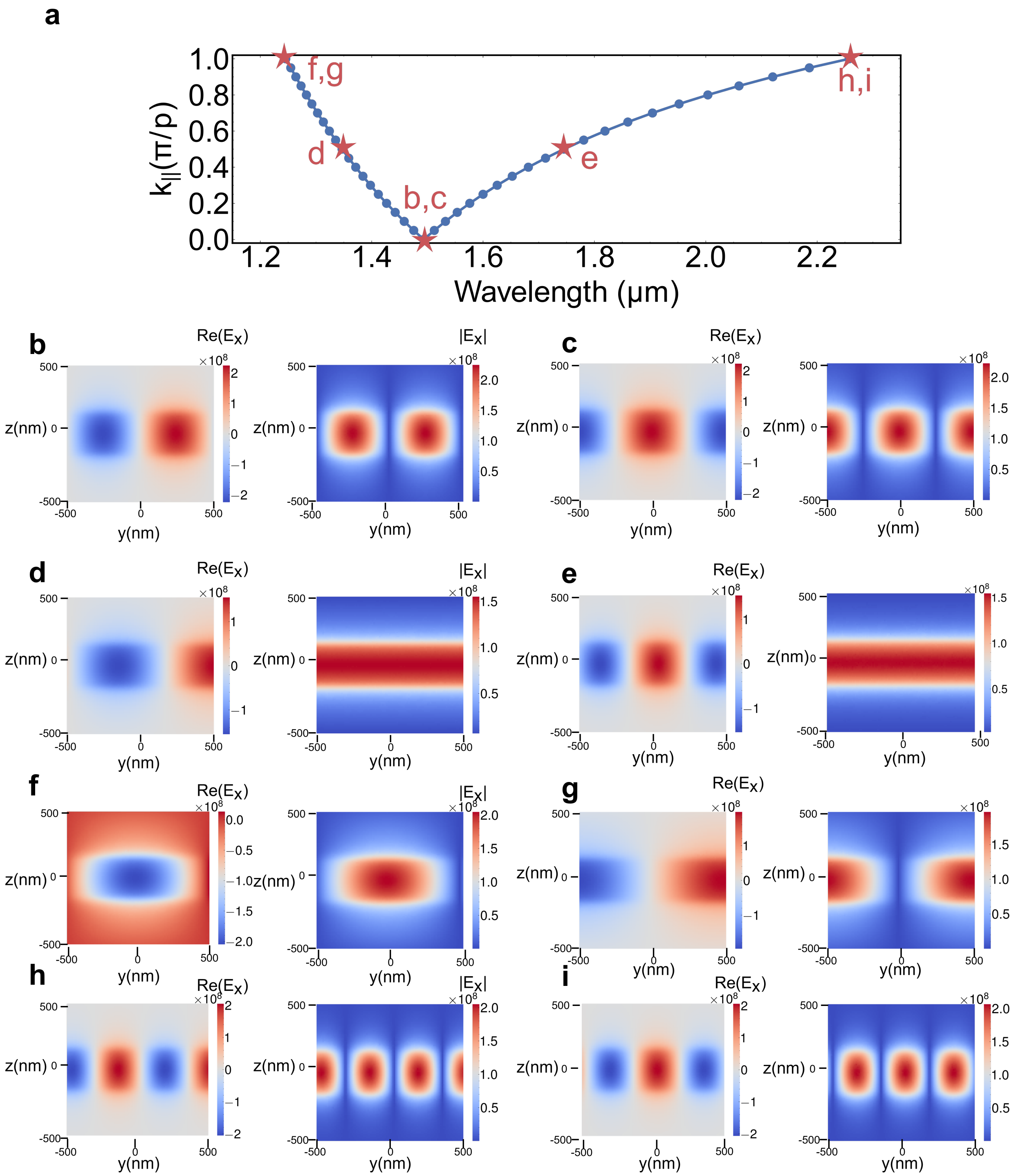

Figure S4 The electrical field profile of slot modes for different $k_{\parallel} = k_y$ in Figure 1b in the main text. a. Redrawing of Figure 1b for mode profile explanation. The red stars label the wavelengths and $k_{\parallel}$ numbers of the mode profile ($Re(E_x)$ and $|E_x|$) in the following subfigures. p: the period of the floquet boundary condition (See Figure 1a). b-c. The mode profile when $k_{\parallel} = 0$. b and c are two degenerate modes. d-e. The mode profile when $k_{\parallel} = \pi/(2p)$. The longer and shorter wavelength mode are described in d and e, respectively. f-i. The mode profile when $k_{\parallel} = \pi/p$. f,g and h,i are two degenerate modes in the longer wavelength end and shorter wavelength end, respectively.

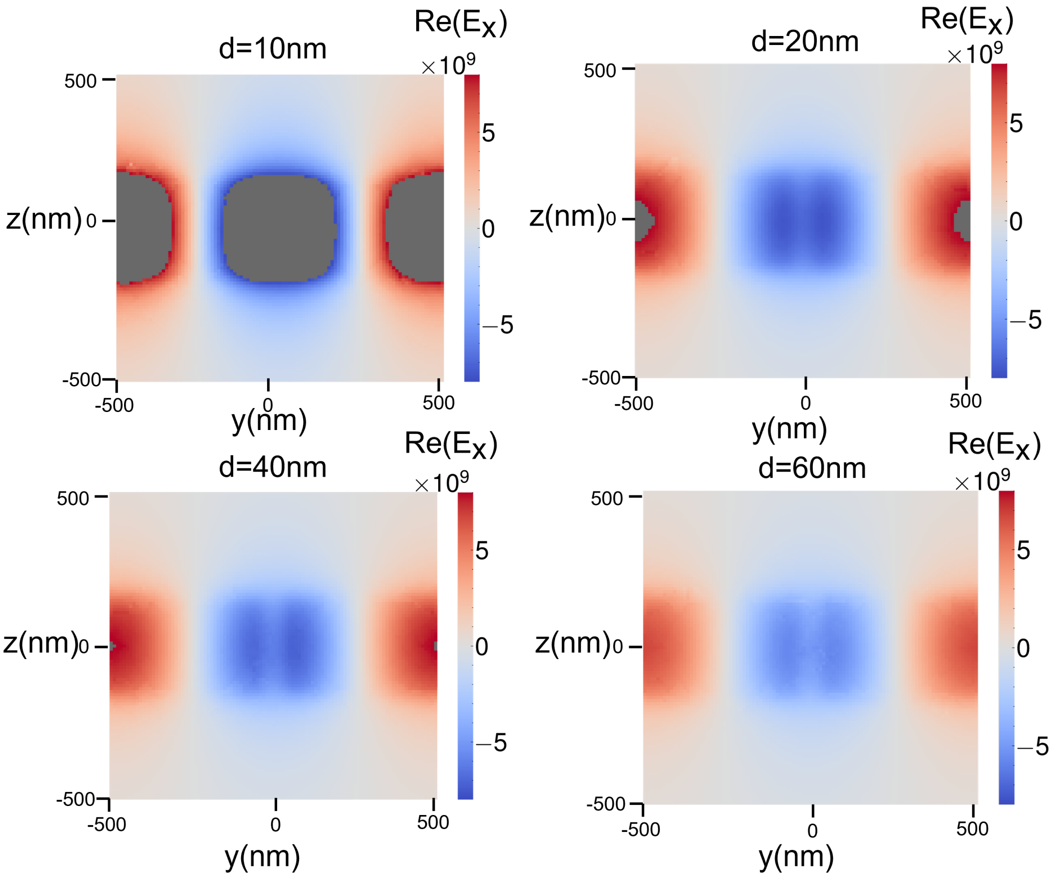

Figure S5 The electrical field profile under normal incidence for different notch sizes in Figure 1c in main text. The profiles are plotted at the resonance wavelength. *d*: The width of the notch perturbation(See Figure 1a). The grey pixels indicate that the field is over or below the limit of the colorbar.

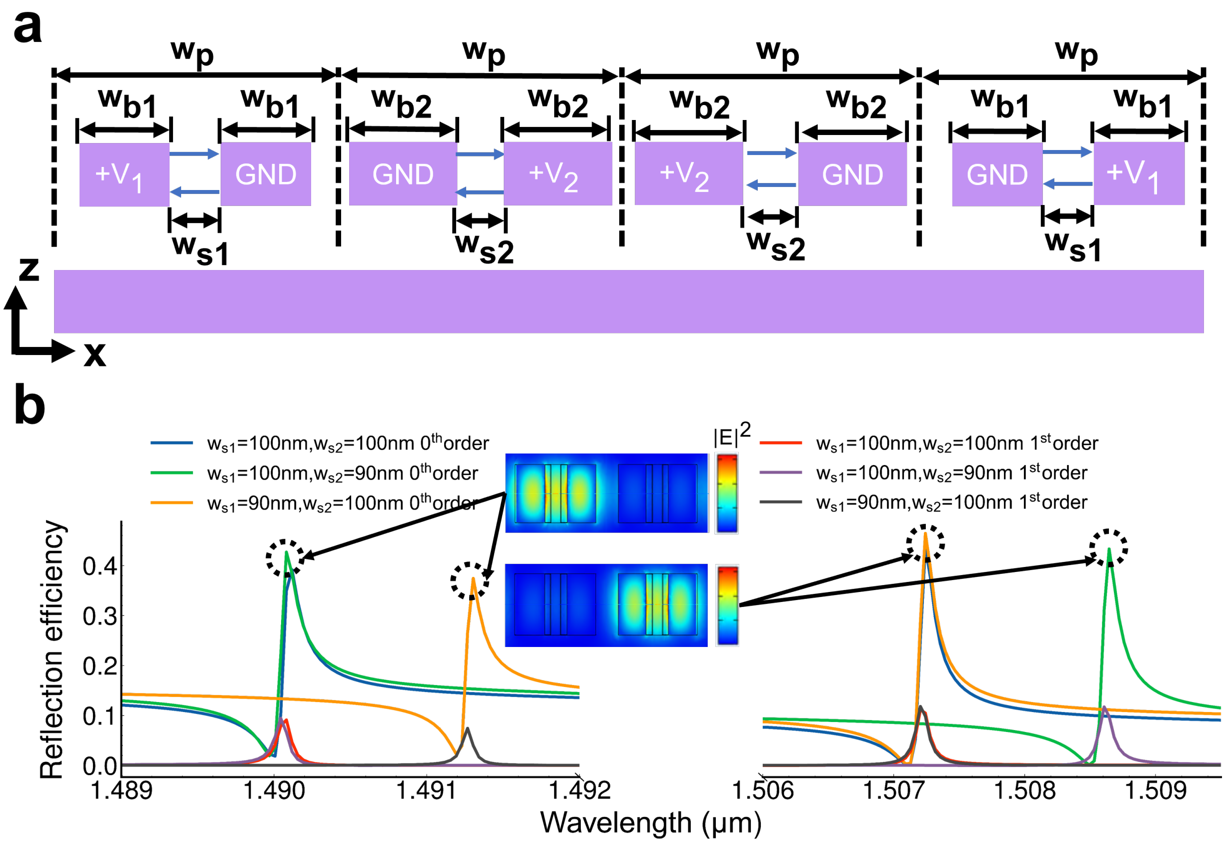

Figure S6 Multiwavelength NEMS tuning. (a) Cross-section of one period for the multiwavelength device. The slot widths where we insert notch perturbations (not shown in the figure) are w_{s1} and w_{s2}, which target the tuning of two wavelengths. Two nanobars that form the slot have the same width w_{b1} and w_{b2} respectively. w_g is the air gap between the grounded bars. The bars that belong to different bias voltages are separated by two ground bars to make sure that two bias voltages will independently control the slot sizes. (b) Spectral shifts due to size changes in different slots. The shrinkage of the slot gap $w_{s1}(w_{s2})$ leads to the redshift of the mode resonance in $1.490\mu m(1.507\mu m)$ while keeping the other mode resonance unperturbed. Insets represent the cross-section of the electric field profile in two slot resonances. The resonance at the lower wavelength represents the resonance in the left slot, and the resonance at the higher wavelength represents the resonance in the right slot.

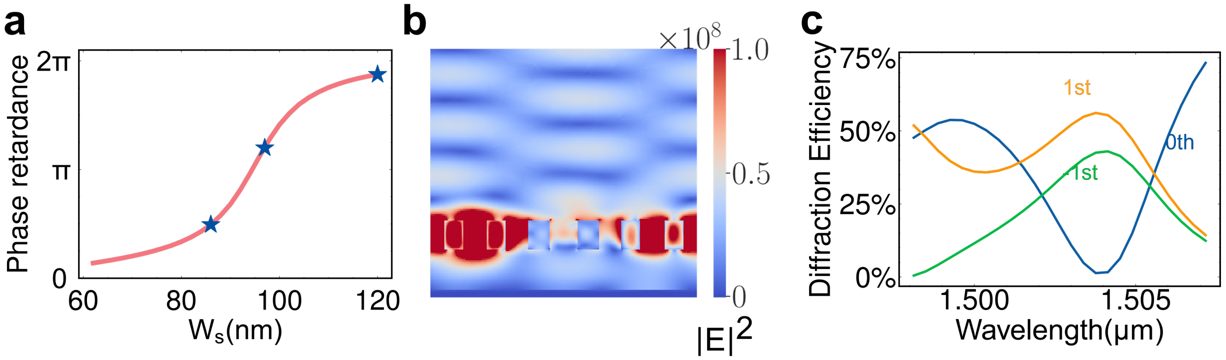

Figure S7 Diffraction test without block. (a) The phase response of the slot mode with respect to the slot size w_s without the blocker. (b) The electric field profile of the device described in (a) at 1.504µm. (c) The diffraction efficiency of the device designed according to (a). Major diffraction orders (0th, 1st, -1st) are shown.
